# News and Coming Events

**Published:** 1909-01-30

**Authors:** 


### News and Coming Events.

Mr. Russell Howard, M.S., F.R.C.S., has been appointed surgeon to the Poplar Hospital.

His Majesty the King has graciously granted permission to Dr. George Ogilvie to accept and wear the Insignia of Knight of the Royal Order of Isabel la Católica, conferred upon him by the King of Spain.

Princess Christian was present at a meeting of the League of Mercy (South St. Pancras District) held at the St. James's Theatre on Thursday, January 28. The speakers included Sir W. J. Collins, M.P., M.D., Captain H. M. Jessel, Mr. H. B. Irving, and the Mayor of St. Pancras.

The death occurred last week in London of Mr. George Eastes, F.R.C.S.Eng., at the age of sixty-seven. Mr. Eastes was for fourteen years chloroformist at the Great Northern Hospital, and had held appointments at Guy's Hospital and elsewhere. He was a past President of the Harveian Society, a member of the Society of Anæsthetists, and a member of the Council of the British Medical Association. Among his contributions to medical literature were a "Memoir of Harvey, the Discoverer of the Circulation of the Blood," and "Evolution in Treatment from 1831 till 1895."

Princess Christian has given her patronage to the *matinée* at the Queen's Theatre, Shaftesbury Avenue, on Thursday, February 18, which is being organised by Lady Constance Hatch, in aid of the funds of the Royal Ear Hospital, Dean Street. Lady Alington and the Hon. Mrs. Edward Stonor have promised to appear in plays, and the Earl of Shaftesbury will sing. Mr. Tom B. Davis will present the principal features of "The Belle of Brittany." A souvenir containing reproductions of photographs, original sketches by eminent artists, and poems and short stories by well-known authors will be provided.

At a meeting of the Central Midwives Board on January 21, the following resolution was carried by five votes to two:—"That the Lord President be respectfully requested to consider the advisability of adding to the departmental committee"—appointed to consider the working of the Midwives Act—"representatives of the interests of general medical practitioners and midwives, as the board consider that such additions would greatly enhance the value of the report eventually come to by that committee." The omission of a direct representative of midwives has led Miss Wilson, as a protest, to resign her seat on the Central Midwives Board, to which she was appointed by the Privy Council.

A course of eight lectures on National Eugenics, in connection with the Galton Laboratory, will be given at University College, London, on Tuesdays at five o'clock, beginning on February 23. The first lecture will be given by Professor Karl Pearson on "The Purport of the Science of Eugenics." On the four following Tuesdays the lectures will be given by Mr. D. Heron, and will deal with the following subjects: Methods of Eugenic Inquiry, Transmission of Physical Characters in Man, Transmission of Psychical Characters in Man, Inheritance of Disease and Deformity. The course will be continued in the Third Term, beginning on May 4, when Miss E. Elderton will lecture on "Effects of Kinship in Marriage," and "Comparison of Heredity and Environmental Factors." Full particulars of the lectures can be obtained from the Secretary of University College, Gower Street, W.C.

Dr. Albert T. Ozzard, of British Guiana, has been elected to the West India Committee.

Lieut.-Colonel J. W. T. Gilbert, V.D., Royal Army Medical Corps (Territorial Force), Third Home Counties Brigade, has received his Majesty's permission to accept the silver medal of the Order of Orange-Nassau, conferred upon him by the Queen of the Netherlands.

By invitation of the Council of the Royal Society of Arts an illustrated lecture was given by Dr. James Cantlie on "The Part Played by Vermin in the Spread of Disease" on Wednesday, January 27, at the Society's rooms, John Street, Adelphi. The chair was taken at eight o'clock by Sir Malcolm Morris.

The Weir Hospital Charity Committee of the Wandsworth Borough Council recommend the Council to pass a resolution that in its judgment it is expedient for the council to prosecute legal proceedings necessary for the promotion and protection of the interests of the inhabitants of the borough in the matter of the Weir Hospital Charity, and to appeal against the scheme of the Charity Commissioners, and to apply the general rate to the payment of the costs and expenses. The contention is that the Weir Bequest of nearly £100,000 was devised to found a hospital at Streatham. The Commissioners intend to devote most of the money to the Bolingbroke Hospital, Wandsworth Common.

The annual general meeting of supporters of the Queen Alexandra Sanatorium, Davos, was held at the Grand Hotel and Belvedere, Davos-Platz, on January 20. Dr. W. R. Huggard, H.B.M. Consul, chairman of the board of management, who presided, announced that Lord Balfour of Burleigh, president of the institution, had recently received from Lord Strathcona a donation of £2,000. It is, however, still necessary to raise another £6,000 in order to be able to open the sanatorium free of debt in the autumn. Dr. Huggard stated that the King had given special permission for the Royal Arms to be placed on the building in stone.

The Chelsea Hospital for Women again shows a record of a successful year's work. Of 720 patients admitted last year, 376 underwent major operations, and the rate of mortality in all cases was under 2 per cent., the figures being almost identical with those of 1907. A prominent feature of the year's work has been the treatment of many cases of cancer of the uterus by the "Wertheim" method. The death of the President, the late Lord Glenesk, towards the end of the year deprived the hospital of an invaluable friend, whose loss it will be very difficult to replace. The annual subscription list for the past year shows an improvement on that of 1907, but in legacies and larger donations there has been a regrettable falling-off.

The St. Petersburg correspondent of *The Times*, writing with reference to the outbreak of cholera, states that on the occasion of the feast of the Epiphany, when the clergy bless the waters of the Neva and the faithful provide themselves with a supply for use in sickness, orders were given by the Metropolitan to various churches to provide receptacles containing boiled water which they were to sanctify and distribute. In consequence of these orders there was an extraordinary scene at the riverside to-day. The customary processions approached the ice, but no one was permitted to descend. The clergy solemnly blessed the river, but they dipped their crosses in copper cauldrons filled with freshly boiled water, which the worshippers afterwards poured into bottles to take home.

The London Provident Dispensaries Council has unanimously passed the following resolution :—"That in the event of the London County Council applying to the London Provident Dispensaries Council to undertake the medical treatment of school children, the Provident Dispensaries Council would do everything possible to meet the requirements of the educational authorities."

The annual meeting of the After Care Association for poor persons discharged recovered from asylums for the insane will be held at 26 Devonshire Place, W., on Wednesday, February 3, 1909, at 3 p.m., when the chair will be taken by Dr. G. H. Savage. After the usual business a paper will be read by Dr. Robert Jones on "The Urgent Necessity of Helping Mental Convalescents." Tea and coffee at 4.15.

The Therapeutical and Pharmacological Section of the Royal Society of Medicine will meet on Tuesday, February 2, 1909, at 4.30 p.m., at 20 Hanover Square, W. Dr. James Mackenzie will read a paper on "Counter Irritation," and Dr. E. I. Spriggs on "The Treatment of Gastric Ulcer, with analysis of cases treated by the Lenhartz method."

Four lectures will be delivered on Tuesday, February 9; Wednesday, February 10; Thursday, February 11; and Friday, February 12, 1909, at Gresham College, Basinghall Street, E.C., by F. M. Sandwith, M.D., Gresham Professor of Physic. The lectures are free to the public, and begin each evening at six o'clock. Lecture I. will deal with "The Results of recent Research on certain Diseases"; Lecture II. with "Diphtheria"; and Lectures III. and IV. with "The Life-work of Pasteur."

The third annual dinner of past and present students of the Royal London Ophthalmic Hospital will be held at the Trocadero Restaurant, Shaftesbury Avenue, London, W., on Wednesday, February 10, under the presidency of Sir Anderson Critchett, Bart., C.V.O. Further particulars can be obtained from the hon. secretaries, Mr. Arnold Lawson, 12 Harley Street, W., and Mr. J. Herbert Parsons, 27 Wimpole Street, W.

The British Association for the Advancement of Science meets this year at Winnipeg from August 25 to September 1. Professor Sir J. J. Thomson, F.R.S., is the president-elect; the president of the section of physiology is Professor Starling, F.R.S.; of the section of botany, Lieutenant-Colonel D. Prain, F.R.S., I.M.S., director of the Royal Botanic Gardens, Kew; and of anthropology, Professor J. L. Myres, of Liverpool.

In view of the number of workmen who have recently been granted superannuation allowances on the grounds of permanent infirmity of mind or body after being in the service of the Kensington Borough Council for a limited number of years, the Finance Committee will, on the suggestion of the Works Committee, recommend that authority to pass a resolution requiring, as a preliminary to the passing of a resolution appointing any workman for the purposes of its Superannuation Act, the submission of a certificate from one of the medical practitioners acting for the council to the effect that he is not suffering from any organic disease and is in all bodily respects fit for the discharge of the work required of him. It is also suggested that the council should pay the necessary fees in connection with the proposed medical examinations.

